# The Dead Presidents

**Published:** 1858-02

**Authors:** 


### THE DEAD PRESIDENTS.

ONLY one third of our Fifteen Presidents are living. Seven of the ten died in the months of June and July, averaging their seventy-fifth year: their average age on entering office was over fifty-seven years. To maintain this average, Mr. Van Buren having passed it two years ago, Mr. Buchanan should live eight years longer, and Messrs. Fillmore, Pierce, and Tyler, about double that time.

Nearly all our Presidents have been men of vigorous health, of hardy constitutions; this is instructively suggestive of the connection there is between high health and the ability to achieve the distinctions to which these men arrived. And investigation will show that the most powerful minds of the last half century were minds which had the substratum of a vigorous constitution, showing that the cultivation of the health of the young is as essential to high success in life as the cultivation of the mind; and, of these, the health should be first, as witness the farm-boys Clay, and Jackson, and Marcy, and Webster, and others like them.

